# Modelling of Protein Kinase Signaling Pathways in Melanoma and Other Cancers

**DOI:** 10.3390/cancers11040465

**Published:** 2019-04-03

**Authors:** Manfred Kunz, Julio Vera

**Affiliations:** 1 Department of Dermatology, Venereology and Allergology, University of Leipzig, Philipp-Rosenthal-Str. 23, 04103 Leipzig, Germany; 2 Laboratory of Systems Tumor Immunology, Department of Dermatology, Universitätsklinikum Erlangen and Friedrich-Alexander-Universität Erlangen-Nürnberg, Hartmannstr. 14, 91052 Erlangen, Germany; julio.vera-gonzalez@uk-erlangen.de

**Keywords:** mitogen-activated protein kinase signaling, *BRAF* oncogene, targeted cancer therapy, checkpoint inhibitors, feedback regulation, systems biology

## Abstract

Melanoma is a highly aggressive tumor with a strong dependence on intracellular signaling pathways. Almost half of all melanomas are driven by mutations in the v-Raf murine sarcoma viral oncogene homolog B (*BRAF*) with *BRAF*V600E being the most prevalent mutation. Recently developed targeted treatment directed against mutant BRAF and downstream mitogen-activated protein kinase (MAPK) MAP2K1 (also termed MEK1) have improved overall survival of melanoma patients. However, the MAPK signaling pathway is far more complex than a single chain of consecutively activated MAPK enzymes and it contains nested-, inherent feedback mechanisms, crosstalk with other signaling pathways, epigenetic regulatory mechanisms, and interacting small non-coding RNAs. A more complete understanding of this pathway is needed to better understand melanoma development and mechanisms of treatment resistance. Network reconstruction, analysis, and modelling under the systems biology paradigm have been used recently in different malignant tumors including melanoma to analyze and integrate ‘omics’ data, formulate mechanistic hypotheses on tumorigenesis, assess and personalize anticancer therapy, and propose new drug targets. Here we review the current knowledge of network modelling approaches in cancer with a special emphasis on melanoma.

## 1. Introduction

Melanoma is one of the most aggressive malignancies with a high mutational burden [1]. This is in part due to a large number of ultraviolet (UV)-induced mutations of a tumor that most often arise from intermittently or chronically sun-exposed areas [2]. However, apart from these mostly silent mutations a number of mutations have been identified that are currently regarded as oncogenic, or driver mutations, that have fostered large studies on small molecule inhibitors that may be used to target the protein products of mutated oncogenes or mutationally activated oncogenic pathways [3]. By far the most important mutated oncogene in melanoma is the v-Raf murine sarcoma viral oncogene homolog B (*BRAF*), with the *BRAF* V600E mutation being the most relevant mutation representing 40–50% of all mutated melanomas and 80% of the *BRAF*-mutated tumors [4,5,6]. The second most important mutated oncogene is the neuroblastoma RAS viral oncogene homolog (*NRAS*) which is mutated in close to 30% of all melanomas [4,5,6]. More recently discovered mutations in the neurofibromin 1 (*NF1*) gene account for 10% of all melanomas [5]. One common theme of all these three mutations is that they are involved in signaling through the mitogen-activated protein kinase (MAPK) pathway, a central pathway in oncogenic signaling [7,8]. The *BRAF* V600E mutation, which was indeed one of the first mutations discovered in melanoma has led to the discovery of specific BRAF inhibitor vemurafenib [9,10,11]. Further specific BRAF inhibitors have gained approval for treatment of BRAF-mutant melanoma such as dabrafenib and encorafenib [3,12]. A further improvement in patient treatment regarding response rates and overall survival came with a combination of BRAF inhibitors and inhibitors of BRAF downstream kinases mitogen-activated protein kinase kinases 1/2 (MAP2K1/2), also termed MEK1/2, which are inhibited by allosteric inhibitors such as cobimetinib, trametininb, and binimetinib [3,12,13]. Combination therapy addressing the MAPK pathway is now a mainstay of targeted melanoma therapy [3].

Since melanoma is a highly immunogenic tumor, activation of the immune system towards melanoma cells has been a major aim of modern melanoma therapy in recent years [3,14,15]. While most cell-based therapies, e.g., via activated pulsed dendritic cells or adoptive T cell transfer have failed so far (clinical trials are still ongoing), two molecules expressed by naive and activated T-cells stand out as targets in more recent studies, cytotoxic T-lymphocyte 4 antigen (CTLA-4) and programmed death 1 (PD-1) [14]. T cell activation needs the activity of co-stimulatory molecules such as CD28 on T cells, bound and activated by B7-1/2 molecules (CD80/CD86) on antigen-presenting cells and tumor cells. However, over-activation of this pathway in T cells is avoided by the expression and activation of the so-called checkpoint molecules CTLA-4 and PD-1, which interact with B7-1/2 and PD-L1 or PD-L2 binding, respectively. While anti-CTLA-4 antibody treatment leads to immune activation in central lymphoid organs, anti-PD-1 or anti-PD-1L antibodies reactivate (normalize) peripheral tumor immunity in the tissue microenvironment [15]. This knowledge has finally led to a new immune-based therapeutic approach using monoclonal antibodies directed against CTLA-4 and PD-1/PD-L1, which have been approved for a variety of different cancers such as melanoma, lung cancer, head and neck cancer, and renal cell carcinoma, with ongoing research to find new immune targets [16]. Treatment response rates differ between both approaches, mainly because of the different modes of action and impact and central versus peripheral immune modulation.

Interestingly, a combination of both, targeted and immunotherapy, appears to be the most promising approach at the moment and clinical trials are ongoing, combining vemurafenib and cobimetinib with atezolizumab, a monoclonal antibody against PD-L1 in melanoma [17]. Overall there are currently more than 1000 clinical trials ongoing combining immunotherapy approaches with different other treatment modalities including targeted therapy, chemotherapy and radiotherapy (www.clinicaltrials.org). However, despite using either targeted treatment or immune-based therapies, recurrence rates are still high and affect the vast majority of patients. This may in part be due to the fact that the complex interplay between different pathways, reactivation of transcriptomic patterns and tumor heterogeneity are poorly understood up to now. 

In the present review, we put an emphasis on oncogenic signaling in cutaneous melanoma, pathway interactions, ‘omics’ data and putative mechanisms of treatment resistance and Systems Biology approaches that may help to understand these mechanisms, with references to some other tumor entities.

## 2. Principles of Mitogen-Activated Protein Kinase (MAPK) and Cellular Homolog of v-Kit Hardy-Zuckerman 4 Feline Sarcoma Viral Oncogene (c-KIT) Signaling in Melanoma

### 2.1. BRAF Signaling

**Figure 1:**
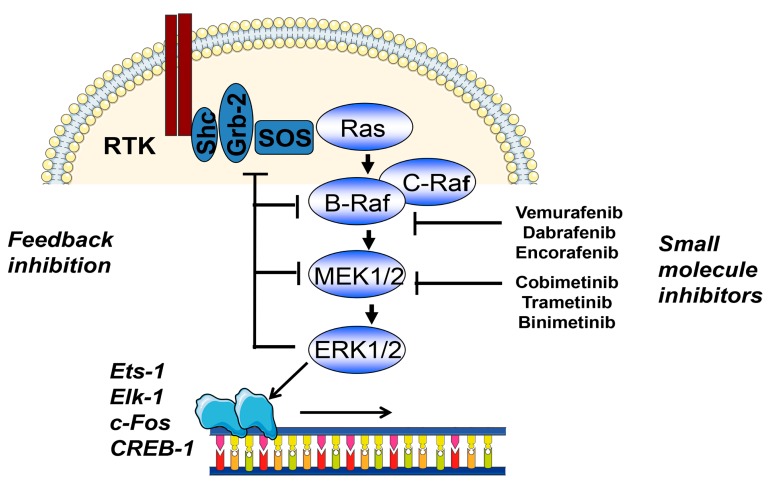
Schematic representation of the MAPK pathway in melanoma. Neuroblastoma RAS viral oncogene homolog (*NRAS*) and v-Raf murine sarcoma viral oncogene homolog B (*BRAF*) are the most commonly mutated oncogenes in melanoma and are strong activators of the MAPK pathway. Within this pathway negative feedback occurs towards mitogen-activated protein kinase kinase 1/2 (MEK1/2), BRAF and son of sevenless homolog (SOS). BRAF and MEK1/2 may by targeted by specific small molecule inhibitors. The activated MAPK pathway further activates transcription factors such as Ets-1, Elk-1, c-Fos, and CREB-1. Abbreviations: RTK, receptor tyrosine kinase; Shc, Src homology 2 domain containing adaptor protein; Grb-2, growth factor receptor bound protein 2; SOS, son of sevenless homolog. Ets-1, V-Ets avian erythroblastosis virus E26 oncogene; Elk-1, ETS-like gene 1; c-Fos, Fos proto-oncogene, AP-1 transcription factor subunit; CREB-1, CAMP responsive element binding protein 1.

The most common BRAF mutation *BRAF* V600E is a strong activator of the MAPK signal transduction pathway (Figure 1). The serine/threonine kinase BRAF acts directly upstream of the MAP2K1/2, also termed mitogen-activated extracellular signal-regulated kinases 1/2 (MEK1/2), which then activate extracellular signal-regulated kinases 1/2 (ERK1/2) [7,8,18]. Early targeting approaches addressing this pathway using sorafenib were not successful, but recently developed more specific BRAF inhibitors led to high response rates, leading to a median overall survival of 24 months when combined with MEK1/2 inhibitors and response rates of up to 65%. Furthermore, much less frequent mutations, such as *BRAF* V600K and *BRAF* V600R, also respond to specific BRAF inhibitors. Interestingly, combination treatment with MEK1/2 inhibitors reduces side-effects that have been linked to paradoxical MAPK pathway activation via CRAF by BRAF inhibitors in normal cells carrying the wildtype *BRAF* allele [19]. This paradoxical activation of the RAS/RAF pathway in case of wild type *BRAF* leads to the development of keratoakanthomas and squamous cell carcinomas in a significant percentage of cases. The underlying mechanisms are not completely understood, but involve conformational changes of the wild type BRAF molecule which then associates with and activates CRAF and the downstream MAPK kinase pathway [20]. 

Interestingly, there is a third mechanism of BRAF activation in case of kinase-mutated, impaired BRAF activity [21]. These specific mutants bind better than wild type BRAF to upstream RAS–guanosine-5’-triphosphate (GTP) with subsequent strong activation of wildtype CRAF leading to increased ERK activity. ERK activation by these mutants is less efficient than that by classical activation of BRAF mutants and thereby induces insufficient feedback to inhibit RAS.

According to more recent *next-generation* sequencing studies, mutations in the *BRAF* gene are associated with mutated up- or downstream molecules which act as tumor suppressors, e.g., cyclin-dependent kinase inhibitor 2A (*CDKN2A*) (p16, p14ARF), *TP53*, and phosphatase and tensin homolog (*PTEN*) [4,5]. Roughly 20% of melanomas have mutations in the *CDKN2A* gene, and more than 10% mutations of *p14ARF* (a splice variant of *CDKN2A*). *TP53* and *PTEN* mutations are present in 19% and 12%, respectively, of melanomas [5]. Their role in different experimental mouse model for melanoma development has been shown in the past, and p16 mutations have been associated with rare familial melanoma cases [22,23,24].

### 2.2. RAS Signaling

*NRAS* is second in the percentage of recurrently mutated genes in melanoma and accounts for close to 30% of mutated melanomas in a mutually exclusive way with *BRAF* [5]. There are, however, cases where mutations in both genes may co-occur, which may be due to the presence of different subclones in an individual sample, which may impact on treatment response and resistance [25]. Kirsten rat sarcoma viral oncogene homolog (*KRAS*)and Harvey rat sarcoma viral oncogene homolog (*HRAS*) may also be mutated in melanoma, but only in a very small percentage of cases (~1%). Activated NRAS further activates downstream effectors such as BRAF and phosphatidylinositol 3-kinase (PI3K) and thereby contributes to tumorigenesis. NRAS has been largely regarded as an undruggable target [26]. Indeed, interference with protein farnesylation, a major mechanism for membrane targeting and activation of NRAS, as well as inhibition of SOS1-mediated nucleotide exchange of RAS, were without success in clinical settings [26]. This might be due to the fact that other mechanisms are active either alone or in combination with these mechanisms that might compensate for this targeted inhibition [27]. RAS targeting might work via inhibition of activated lysine methyltransferases, as has been shown for pancreatic cancer [28]. The underlying mechanism involves the inhibition of lysine methyltransferase SET and MYND domain Containing 3 (SMYD3), which acts on MAP3K2 at lysine 260. 

NRAS-mutated melanomas were shown to respond to MEK1/2 inhibition in a small percentage of cases, which has, however, not led to an approval for treatment of melanoma patients carrying *NRAS* mutations. For example, binimetinib, the most recent approved MEK1/2 inhibitor for melanoma treatment for combination therapy with a BRAF inhibitor, was able to induce at least a partial response in 20% of melanoma patients with *NRAS* mutations [29]. Overall, there are no currently available small molecule inhibitors that have reasonable activity in *NRAS*-mutated melanomas. Currently used downstream MEK1/2 inhibitors target wild type MEK1. Since MEK1/2 are mutated in a small percentage of melanomas this further adds to the challenge of treating *NRAS*- and MEK1/2-mutated melanomas.

Oncogenic signaling through RAS (NRAS, HRAS, or KRAS) has been in the focus of recent research because they are often mutated in a variety of cancers and have been widely regarded as undruggable [26]. This is in part due to the fact that the activation pathway has not been clear-cut in the past. In a recent report, it was demonstrated that overexpressed and mutated, and thereby activated, KRAS localized to the cell membrane and formed dimers with subsequent activation (phosphorylation) of ERK1/2 [7]. By introducing an artificial dimerization domain into the KRAS construct and addition of a small molecule that supported dimerization it could be further shown that dimerization and activation were independent of overexpression of the oncogene. Moreover, a mutant CAAX (C, cysteine; A, aliphatic amino acid; X, any amino acid) RAS membrane-targeting domain abolished MAPK pathways activation. Together, these experiments showed that both membrane targeting and dimerization is important for RAS activation apart from the known binding of GTP.

### 2.3. Signalling through Receptor Tyrosine Kinases

The receptor tyrosine kinase (RTK) c-KIT is recurrently mutated in a small percentage of melanomas [5]. However, its prevalence in mucosal and acral melanomas is significantly higher (up to 20%) [30]. Mutations are located in the intracellular juxtamembrane domain and two intracellular kinase domains. The most prevalent mutation *KIT* L576P, which was found in 30% of all cases leads to downstream activation of both MAPK and PI3K/Akt pathways. Although initial studies have shown some response to KIT inhibitor imatinib for KIT L576P and KIT K642E, later larger clinical trials were disappointing, including other KIT inhibitors such as dasatinib or nilotinib [31]. Overall, in contrast to gastrointestinal tumors, c-KIT appears at present not to be a promising target in melanoma. RTKs are mainly involved in the development of tumors of epithelial origin and hematological malignancies [32]. The family of RTKs comprises 20 subfamilies with a total of 58 members. Genetic aberrations include mutations, deletions, translocations, and amplifications. Well-known examples of mutated oncogenic members are found in the epidermal growth factor receptor/Erb-B2 receptor tyrosine kinases (*EGFR/ERBB*) family involved in lung adenocarcinoma and other non-small cell lung carcinomas, breast cancer, and colorectal and gastric cancer. In melanoma, *ERBB2* mutations have been recently described in a small percentage of 1% of cutaneous melanomas [33]. Secreted ERBB3 has been shown to support metastasis in melanoma without being affected by mutations [34]. The platelet-derived growth factor (*PDGR*) family member PDGFRα is mutated in a small percentage of gastrointestinal stroma tumors. Several members of the fibroblast growth factor receptor (*FGFR*) family such as *FGFR1-3* were found to be mutated in squamous cell lung carcinoma, breast cancer, endometrial, gastric, ovarian and urothelial cancers. Melanomas which are wild type for *BRAF*, *NRAS,* and *NF1* show amplifications of *KIT*, platelet-derived growth factor receptor α (*PDGFRA*)*,* and vascular endothelial growth factor receptor 2 (*VEGFR2)* in 10–20% of cases [5]. For a more detailed view on this group of oncogenic signaling molecules the reader is referred to the above mentioned recently published comprehensive review [32].

## 3. Signaling through Other Mutated Pathways

**Figure 2:**
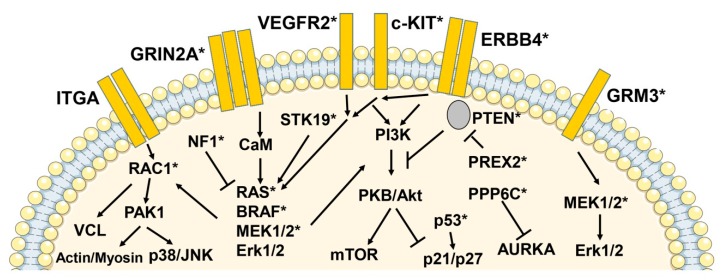
Schematic representation of signaling pathways with activating and inactivating mutations or gene amplifications in melanoma. In large-scale sequencing studies using *next-generation* sequencing a number of activating and inactivating mutations and gene amplifications of genes encoding for intracellular signaling molecules could be identified, apart from *BRAF* and *NRAS*. A schematic representation of these molecules and pathways is shown. Protein products of mutated or amplified genes are indicated by asterisks. Abbreviations: ITGA, integrin alpha; GRIN2A, glutamate [NMDA] receptor subunit epsilon-1; VEGFR2, vascular endothelial growth factor receptor 2; KIT, v-Kit Hardy-Zuckerman 4 feline sarcoma viral oncogene homolog; ERBB4, Erb-B2 receptor tyrosine kinase 4; GRM3, glutamate receptor, metabotropic 3; RAC1, Ras-related C3 botulinum toxin substrate 1; NF1, neurofibromin 1; STK19, serine/threonine-protein kinase 19; PI3K, phosphatidylinositol 4,5-bisphosphate 3-kinase; PKB, protein kinase B; PREX2, phosphatidylinositol-3,4,5-trisphosphate-dependent Rac exchange factor 2; PTEN, phosphatase and tensin homolog; PAK, p21-activated kinase 1; CaM, calmodulin; mTOR, mechanistic target of rapamycin; MAP2K1/2 (MEK1/2), mitogen-activated protein kinase kinase 1/2; Erk1/2, extracellular signal-regulated kinase 1/2; JNK, c-Jun N-terminal kinase, VCL, vinculin; PPP6C, protein phosphatase 6 catalytic subunit; AURKA, aurora kinase A.

Apart from NRAS, BRAF, and KIT, there is a series of further recurrent mutations in other pathways (Figure 2). The impressing number of currently available sequencing studies has consolidated our knowledge of the melanoma mutational landscape [4,5,6,35]. Melanoma is one of the tumors with the highest mutational burden (more than 10 non-synonymous mutations per megabase per tumor) [1]. In addition to the above mentioned genes, earlier reports of melanoma high-throughput sequencing studies provided some additional candidates for melanoma oncogenes. Evidence was provided for mutations in matrix metalloproteinases (MMP8), receptor tyrosine kinases and or G-protein coupled receptors (*GPCR*) [36,37,38,39]. GPCRs are also involved in resistance to BRAF and MEK1/2 inhibitors [40]. GPCRs with a role in melanoma biology appear to be melanocortin type 1 receptor, endothelin receptor, metabotropic glutamate receptors-1, 3, and 8, and platelet-activating factor receptor [37,39]. The ionotropic glutamate receptor *GRIN2A* and the metabotropic glutamate receptor *GRM3* were found to be mutated in 25% and 16%, respectively, of melanoma samples, but without recurrent mutations for *GRIN2A* and with only a small percentage of recurrent *GRM3* mutations. The clinical significance of these findings remains to be determined.

One of the most challenging findings was the detection of mutations in the *ERBB4* gene [36]. *ERBB4* was mutated in up to 20% of all tested samples in this study, but showed no recurrent mutations. ERBB4 is a member of the EGFR family, which is known to play a role in experimental melanoma models. However, early clinical trials using the pan-EGFR inhibitor lapatinib were not promising. Moreover, subsequent studies showed that the percentage of *ERBB4*-mutated samples was indeed much lower in an independent cohort (around 3%), and the higher percentage found in the first study might have been due to a sampling bias of melanoma cell cultures versus melanoma tissues [41]. 

*PREX2*, a RAC exchange factor, has been found to be mutated in 14% of melanoma samples, again without recurrent mutations [42]. Although some functional data in a mouse model showed that mutant *PREX2* made immortalized melanocytes more aggressive affecting the survival rates of melanocyte-injected mice, the functional role of mutated *PREX2* remains elusive. Finally, *RAC1* was found to be recurrently mutated at the same position (*RAC1* P29S) in melanoma samples in three independent studies [4,5,43] with a mutation rate of 5%. *RAC1* is a member of the Rho Family of small GTPases. Functionally, mutated RAC1 may increase the proliferation of melanoma cells. However, small molecule inhibitors against RAC1 have not been tested in clinical trials up to now, which might be due to the relatively small percentage of melanoma patients affected by this mutation.

Overall, the top 10 mutated genes in the mentioned study of the Cancer Genome Atlas Network were *BRAF, NRAS, PTEN, NF1, TP53, CDKN2A, RAC1, ARID2, DDX3X,* and *PPP6C,* with only *BRAF, NRAS, RAC1,* and *PPP6C* showing recurrent mutations [5]. In addition, serine threonine kinase *STK19*, which phosphorylates and thereby activates NRAS, has been shown to be recurrently mutated in melanomas in an earlier study [4]. This further emphasizes the fact that the activated MAPK pathway may indeed be the most relevant mechanism for melanoma biology.

Some new aspects were provided by sequencing of the upstream region of the telomerase reverse transcriptase (*TERT*) gene [41]. *TERT* promoter variations were found in 33% of primary melanomas and 85% of metastatic lesions, but were distributed over several different positions [44]. *TERT* mutations are an early event in melanoma development. So-called intermediate lesions between benign precursor lesions and invasive melanomas were shown to be positive for *TERT* mutations in close to 80% [45]. 

## 4. Treatment Resistance to MAPK Inhibition

The vast majority of cases with treatment resistance, either as primary resistance or secondary resistance, involve MAPK and PI3K pathways. In one of the first studies that addressed this issue using *next-generation* sequencing technology, it was shown that the genetic aberrations of vemurafenib resistance included *NRAS* mutations, *BRAF* amplifications, *MEK1/2*, *PIK3CA,* and *PTEN* mutations, as well as microphthalmia-associated transcription factor (*MITF*) amplifications [46]. Subsequent in vitro assays showed that treatment of *BRAF*-mutant *PTEN*-deficient melanoma cells with a combined vemurafenib and PI3K inhibitor was more efficient regarding anti-proliferative activity than single-agent treatment. 

In a study that used a high-throughput knockdown screen of 15,000 genes, a series of G-protein coupled receptors was shown to mediate resistance against BRAF-, MEK1/2- or ERK1/2 inhibitor treatment in melanoma cell lines [40]. The downstream signaling effects of these receptors involved adenylate cyclase, cAMP, protein kinase A (PKA), and finally *MITF* gene transcription. Resistance could be overcome by use of histone deacetylase inhibitors that inhibited the MITF gene transcription and thereby MITF-induced resistance.

In contrast to these findings, resistance may also be conferred by a MITF-low expression phenotype as determined by two recently published studies [47,48]. In the first study, it was demonstrated that primary treatment resistance against BRAF and MEK1/2 inhibitors was associated with a MITF-low/NF-κB-high phenotype with reduced expression of MITF, melan-A (*MLANA*), premelanosome protein (*PMEL*), tyrosinase-related protein 1 (*TYRP1*), neuropilin 1 (*NRP1*), and enhanced expression of *AXL*. These findings further support the notion that MITF may by pro-tumorigenic at high and low expression levels. Similar results were obtained in the second independent study that identified an explicit MITF-low/AXL-high phenotype [48].

The tumor microenvironment may also confer resistance to MAPK inhibitor treatment by secretion of macrophage-derived tumor necrosis factor-α (TNF-α) [49]. It was suggested that the TNF- mediated on-treatment resistance in melanoma cells is mediated by IκB kinase IKK signaling and MITF expression. However, evidence has also been provided that TNF may downmodulate MITF expression and TNF per se may induce a de-differentiated phenotype [50,51]. Overall, the impact of MITF-high or MITF-low phenotypes on MAPK resistance is still a matter of debate [52].

In a PCR-based resistance screen to dabrafenib or vemurafenib, *BRAF* splice variants, *MEK1/2*, *NRAS*, and *AKT1* were sequenced [53]. More than half of all patients showed *BRAF* splice variants, *NRAS* mutations, *BRAF* amplifications, and *MEK1/2* mutations under treatment resistance, and 80% of patients showed a re-activation of MAPK signaling as determined by expression analysis of MAPK gene signatures.

Mechanisms of double drug resistance (vemurafenib/cobimetinib) included *BRAF* amplifications, *NRAS* mutations, *CDKN2A* deletions, and *PTEN* loss-of-function mutations [54]. Double drug resistant cell lines were shown to be drug addicted to both substances, and removal of both drugs led to rapid cell death. Interestingly, drug addiction could be overcome by a triple combination of BRAF and MEK1/2 inhibition together with an ERK1/2 inhibitor. ERK1/2 inhibitor alone recovered cell growth of otherwise dying cells. This might argue for a drug holiday in targeted melanoma treatment but needs further investigations. Drug resistance mediated by pathway over-activation was also found in an earlier study of single-agent vemurafenib-treated melanoma cells [55]. Treatment resistance may involve different developmental trajectories leading to different treatment resistance mutational patterns in one patient [56]. Individual disease-progression biopsies may thus not necessarily indicate global resistance patterns, in line with the clinical observation of mixed tumor responses under targeted treatment.

A combined large-scale study analyzing exome, transcriptome and methylome data revealed a number of classical resistance mutations such as gain-of-function mutations or amplifications of *BRAF V600E/K*, *NRAS*, *KRAS*, *MEK1*, *PIK3CA*, *AKT1*, and *AKT3*, and loss-of-function mutations in phosphoinositide-3-kinase regulatory subunit 2 (*PIK3R2*)*,* dual specificity phosphatase 4 *(DUSP4*)*, CDKN2A*, and *PTEN* in combined treatment (BRAF/MEK inhibition) resistant melanoma cells [57]. Transcriptome analysis revealed a series of up-regulated genes encoding for c-MET, IL-8, and macrophage markers CD163 and CD163L1 in treatment-resistant samples. The different gene expression patterns showed a correlation with methylation at specific CpG clusters. In in vitro experiments, resistance could be reversed by treatment of cells with hepatocyte growth factor receptor (c-MET) inhibitor crizotinib. Moreover, treatment of cells with a glycogen synthase kinase 3 β (GSK3β) inhibitor reactivated b-catenin-lymphoid enhancer binding factor 1 (*LEF1*) expression and re-sensitized melanoma cell lines to BRAF and BRAF/MEK1/2 inhibition. 

Driver mutations (BRAF/NRAS) in primary melanomas and corresponding metastatic lesions are concordant in the vast majority of cases [58]. As mentioned above, driver mutations in post-treatment recurrence appear to be based on a limited number of different molecular mechanisms such an amplification of mutant *BRAF* or a switch to *NRAS* mutations, or mutational activation of the PI3K-Akt pathway [46,54].

It is still a matter of debate, whether treatment resistance is due to overgrowth of pre-existing resistant clones or secondary mechanisms, or both. A recent study used an experimental setting where a pre-existing vemurafenib-resistant tumor cell clone was injected into mice together with a significantly larger number of vemurafenib-sensitive melanoma cells [59]. Under vemurafenib treatment, the resistant cells formed detectable tumors but no tumors under control no-treatment conditions. Resistant melanoma cells activated the PI3K/AKT/mTOR pathway. Inhibition of this pathway reduced tumor growth. A treatment-induced secretome supported tumor growth of the drug-resistant cancer cell clones. Downregulation of cJUN transcription factor family member fos-related antigen 1 (*FRA1*) was associated with the expression of the tumor-promoting secretome for melanoma cells.

## 5. The ‘Omics’ Approach in Melanoma and Treatment Sensitivity

As described above, progression of melanoma and acquisition of resistance phenotypes to pharmacological treatment is a consequence of the deregulation of molecular pathways like MAPK cascades via mutation, gene amplification, abnormal gene expression, or epigenetic changes. However, these pathways do not exert their function in isolation. Rather, they cross-talk to each other by sharing signal mediators or through more complex mechanisms like combined transcriptional regulation, thereby establishing tightly entangled regulatory networks. The deregulation of these networks induces specific cancer phenotypes like malignant cell growth, tumor progression, treatment resistance, and metastasis. 

Large data sets are now available due to the development of high- throughput data generation technologies like microarray technology and *next-generation* sequencing, proteomics, and phosphoproteomics that help to reconstruct and decode these networks. The inspection of these datasets making use of advanced bioinformatics pipelines can be used to find ensembles of genes collectively deregulated, for example, in a population of patients displaying resistance to a given therapy. By inspecting the pathways and cellular processes in which these genes are involved by pathway enrichment analysis, one can associate the patient populations and their gene signature to given, supposedly deregulated pathways. 

In one of the first studies in this field, transcriptomic microarray data were used to identify gene patterns of early melanoma development and tumor progression [60]. In this study, gene expression profiles of non-metastatic and isogenic metastatic A375 melanoma cells were compared. The major difference between both profiles was based on the overexpression of RhoC, a well-known GTPase that activates downstream signaling pathways of the actin-based cytoskeleton. Interestingly, RhoC does not directly signal into the MAPK pathway. In a later study, a series of 31 melanoma samples, mostly metastases, was analyzed using microarray technology [61]. Authors identified two major clusters of melanoma lesions representing different aggressiveness. Gens patterns mainly differed in the expression of adhesion and matrix molecules such as integrin β1, integrin β3, syndecan, vinculin, and fibronectin.

Most of these data in earlier studies were derived from melanoma cell lines or from metastatic lesions. We performed a large-scale transcriptomic analysis by use of microarray technology on microdissected material of tissue biopsies from primary melanomas and metastatic lesions [62]. By this means, we identified a series of overexpressed genes that related to cell cycle activation overexpressed in metastatic lesions e.g., cell division cycle 6 (*CDC6*), cyclin dependent kinase 1 (*CDK1*), septin 6 (*SEPT6*), mitosin (*CENPF*), and kinesin family member 2C (*KIF2C*). Cdc6 and Cdk1 are well-known downstream targets of MAPK signaling. By use of mathematical modelling of transcriptomic data, a performance of more than 85% for correct classifications of primary melanomas and metastases was reached. Gene expression patterns could also distinguish primary melanomas of different thickness, supporting the notion that gene expression profiles may support clinical classifications.

A further transcriptomic analysis was provided by a group that analyzed microdissected fresh frozen material of 18 melanocytic nevi, 20 primary melanomas, and 20 metastatic melanomas using microarray technology [63]. The findings of differentially expressed genes were subsequently verified in a 280 sample tissue microarray. Melanoma samples could be grouped based on gene expression pattern using principle component analysis. Among upregulated genes were preferentially expressed antigen in melanoma (*PRAME*) and osteopontin (*SPP1*), also found in other publications [64], and immune genes such as C-X-C motif chemokine ligand 9 (*CXCL9*), S100 calcium binding protein A9 (*S100A9*), melanoma cell adhesion molecule (*MCAM*), and cell cycle genes such as *CDK2* and *CDK4* [65]. 

In the currently most comprehensive analysis of melanoma transcriptomes, 329 samples were analyzed by RNA-seq analysis [5]. Authors identified three robust clusters termed ‘immune’ (*n* = 168), ‘keratin’ (*n* = 102), and ‘MITF-low’ (*n* = 18) which refers to predominant gene expression signatures in these samples. Patients in the immune cluster had more favorable prognosis than those of the other clusters. The keratin cluster showed high expression of keratin, pigmentation, and epithelial genes, supporting the notion that melanoma cells express at least some keratin genes. In particular, the identification of an immune signature may have consequences regarding currently widely used treatment modalities with immune checkpoint inhibitors. Recently, we performed an RNA-seq study using laser-microdissected tissues from 23 benign melanocytic nevi and 57 primary melanomas [64]. This was indeed the first RNA-seq study that used laser-microdissected tissues in order to largely avoid contamination with epidermal keratinocytes and immune cells of the tissue microenvironment. We identified two transcriptomic types of melanomas with some gene signatures already present in benign nevi. N1/M1 lesions showed pigmentation-type and MITF gene signatures and a high percentage of *NRAS* mutations in M1 melanomas. N2/M2 lesions showed an inflammatorytype similar to the above described immune cluster and an AXL gene signature, with an equal distribution of wild type and mutated *BRAF* and a low prevalence of *NRAS* mutations in M2 melanomas. Analysis by pseudotime dynamics (Wanderlust software) of nevus and melanoma samples showed a switch-like downregulation of immune genes indicative of an immune-escape mechanism in late-stage, highly proliferative melanomas. 

## 6. Systems Biology Approaches Used to Understand Kinase Inhibitor Activity and Resistance: from Networks to Computational Models

**Figure 3:**
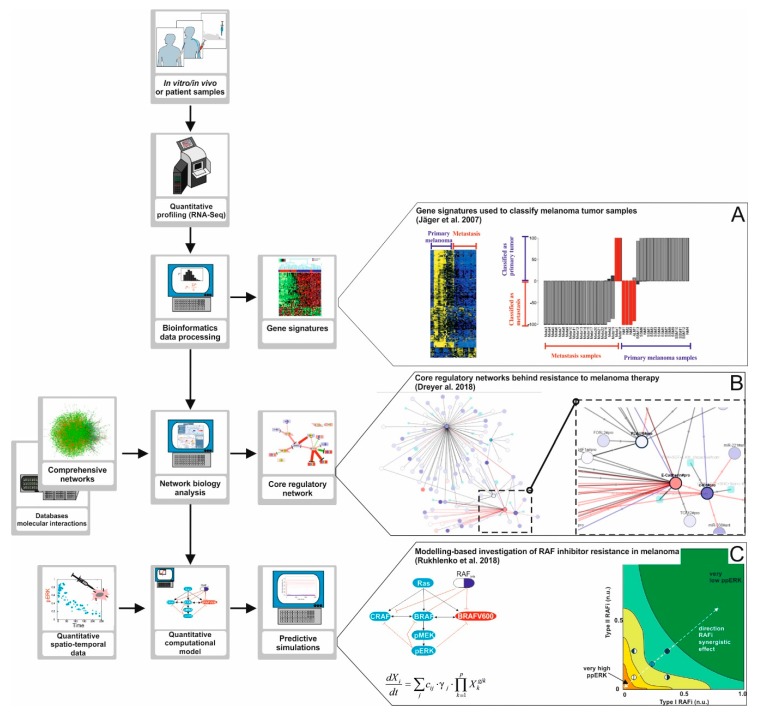
A road map to apply Systems Biology approaches in melanoma research. The development of high-throughput data generation technologies like *next-generation* sequencing, proteomics, and phosphoproteomics opens opportunities to investigate the mechanisms of emergence for BRAF inhibitor therapy resistance in melanoma in depth. (**A**) High-throughput data analysis. The data obtained from the quantitative profiling of tumor samples can be processed and analyzed making use of bioinformatics pipelines and algorithms. Using this approach, one can discover gene signatures, that is, ensembles of genes collectively deregulated in tumor samples from a group of patients. In the study of Jäger and co-workers [62], tumor samples were profiled and the data obtained was used to find a predictive gene signature for metastatic melanoma. (**B**) Network Biology. The processed high-throughput data can also be analyzed using network biology approaches. In this case, the data are mapped into large molecular networks, and computational algorithms are used to detect core regulatory networks, highly interconnected fractions of larger networks that are differentially regulated in, for example, treatment responders versus non-responders. The core network, compared to a gene signature, gives additional information because it helps constructing hypotheses about the molecular mechanisms linking the differentially regulated genes. In a study by Dreyer and co-workers [66] we reconstructed a comprehensive network accounting for signaling pathways commonly deregulated in melanoma. The network was used to detect a core regulatory network discriminating responders from non-responders in a cohort or patients treated with anti-PD-1 immunotherapy. The comprehensive network derived is available at www.vcells.net/melanoma. (**C**) Computational modelling of regulatory pathways. High- throughput data, core regulatory networks, and additional quantitative data like drug dose response curves or time series of signal pathway activation can be used to derive, characterize, and train computational models of regulatory networks relevant for therapy. Computational simulations performed with these models can be used to formulate hypotheses, design experiments, or even to (re)design combinatory therapies. In a study by Rukhlenko and co-workers [67] simulations of a detailed computational model of the RAF/MEK/ERK signaling pathway in melanoma were used to predict combinatory BRAF inhibitor therapy able to re-sensitize therapy resistant melanoma cell lines.

Bioinformatics analysis combined with pathway enrichment can deliver ensembles of genes collectively deregulated in cancer linked to given cancer phenotypes (Figure 3). However, one can go further into the understanding of the mechanisms behind melanoma progression and therapy resistance when the molecular interactions between these and other relevant genes are elucidated making use of network biology approaches. Network biology is a branch of bioinformatics that provides methodologies for the reconstruction of large biochemical networks, as well as for employing these networks for the analysis, interpretation and visualization of high-throughput data sets. 

It is important to understand that the deregulated network behind cancer is both large and tumor entity specific. In line with this, we recently reconstructed a comprehensive network of signaling pathways commonly deregulated in melanoma biology [66], (www.vcells.net/melanoma). The reconstruction was performed using information about molecular factors and interactions extracted from the current literature by manual curation, as well as from public databases by using bioinformatics pipelines.

Together with MAPK pathways, the map includes detailed reconstructions in the context of melanoma, e.g., for, growth factor receptors signaling, TNFα signaling, AKT pathway, p53 and BCL-2-linked apoptosis, WNT and NOTCH pathways, cell cycle regulation, hypoxia-induced signaling, and E-cadherin and integrin-mediated signaling. The resulting network contains 815 molecular species (genes, mRNAs, miRNAs, proteins, and protein complexes) interconnected through 972 reactions. Interestingly, the network reconstruction highlighted the fact that the regulatory pathways included, rather than isolated, are tightly entangled and cross-talk to each other. 

Using this type of networks, the MAPK signaling pathway is put in the context of its interactions with other key melanoma pathways, like growth factor signaling, AKT, and cell cycle regulation, an interaction that we think is critical to understand the emergence of kinase inhibitor resistance. Furthermore, a core regulatory network, deregulated in melanoma patients not responsive to anti-PD-1 therapy, with linked genes related to cell cycle control and epithelial-to-mesenchymal transition was identified.

Using computational algorithms, these networks can be utilized to mine and visualize in vitro and patient high-throughput data sets. One possibility is to obtain the so-called core regulatory networks, a highly interconnected fraction of the large network that is differentially regulated in a population of cancer patients, for example, resistant to a given therapy. The genes contained in the core regulatory networks are supposed to account for an ensemble of molecularly interlinked markers for the condition investigated, and the network provides insights into the molecular mechanisms for this association [68]. In Dreyer et al. 2017, we used the comprehensive network derived to map and analyze tumor RNA-Seq data from a cohort of melanoma patients under anti-PD-1 immunotherapy. We discovered a core regulatory network composed of 41 molecular factors differentially regulated in responders versus non-responders, twenty of them linked to epithelial to mesenchymal transition (EMT). 

Core regulatory networks can also be used to select targets for a combinatorial drug treatment, or in a drug discovery process. In a study of Zecena and co-workers, a network-based computational pipeline was applied to investigate the mechanisms of resistance to MAPK inhibitors in malignant melanoma [69]. To this end, the authors first developed an experimental model of resistance to vemurafenib by exposing the melanoma cell lines SK-MEL-28 and A375 to incrementally increasing concentrations of the drug. Total tumor RNA from the selected treatment-resistant cell lines were profiled using RNA-seq and their profiles were compared to that of the original, non-resistant SK-MEL-28 cell line. The authors found nearly 1000 genes showing significant differential expression. When they performed a pathway enrichment analysis they found that multiple genes involved in MAPK signaling and EMT were differentially expressed. To substantiate the results, the authors used transcription factor (TF) motif analysis to find the key TFs whose deregulation could explain the transcriptional reprogramming behind the resistance to vemurafenib. The analysis produced a core regulatory network involving interlinked, differentially expressed targets and overexpressed TFs, including NF-κB, zinc finger E-Box binding homeobox 1 (*ZEB1*), integration host factor (*IHF*), and E2F-related TFs. Interestingly, upregulation of ZEB and E2F TFs have been linked to drug resistance in multiple tumors, including melanoma [70,71]. These results emphasize the fact that resistance to kinase inhibitors relies in the deregulation of a network of entangled pathways. 

Network analysis can be employed to integrate and analyze large data arrays from systematic experiments. To investigate the regulation of the key cell survival signaling pathway AKT in breast cancer, Lu and co-workers systematically targeted a large number of kinases and kinase-related factors with small interfering RNAs (siRNAs) [72]. The effect of siRNA-targeting was assessed by proteomics analysis of 42 proteins and phosphoproteins. When making a statistical data analysis, the authors found that siRNAs targeting of a group of 115 genes had an inverse correlation with the phosphorylation of AKT and MAPKs. Next, the authors used pre-existing databases of molecular interactions to reconstruct a large network connecting these genes to AKT and MAPK signaling pathways, and computational algorithms to detect a core MAPK signaling-related regulatory network able to increase AKT phosphorylation upon siRNA targeting. The results suggest that inhibition of MAPK signaling can in some conditions induce the phosphorylation of AKT. Having in mind that phosphoproteomics data has already been produced in the context of AKT activity and melanoma resistance to MAPK inhibitors (vemurafenib and selumetinib), one can envision that a similar approach could be used to detect a core regulatory network behind the interplay between AKT and MAPK cascades in melanoma kinase inhibitor resistance [73].

Biochemical networks deregulated in melanoma and other aggressive cancers contain myriads of cross-talking, intersecting network motifs. These are small regulatory circuits like feedback- and feedforwardloops, which upon deregulation can induce counter-intuitive, unexpected molecular behavior. Mutation of oncogenes can disrupt the exquisite regulation of signaling pathways exerted by negative feedback loops, ERK signaling being a paradigmatic case in point [74,75]. On the other hand, deregulation of positive feedback loops in signaling-mediated circuits can induce self-sufficiency in growth signals. The interplay between these intersecting network motifs embedded in large network is better understood when high-throughput data are investigating making use of computational modelling. A computational model is a set of equations or other mathematical entities encoding the basic properties of the investigated network. In biomedicine its computer simulations can be used to derive hypotheses on molecular mechanisms, design validation experiments, or to boost drug target discovery. Korkut and co-workers made use of high-throughput data from RAF inhibitor (RAFi)-resistant melanoma cells (SK-Mel-133 cell line) and computational network modelling to investigate escape mechanisms behind the resistance to RAF inhibitor combinations in aggressive melanoma [76]. The melanoma cells were challenged with combinations of targeted drugs and the changes induced in proteins (88), phosphoproteins (50), and cell phenotypes (5) were quantified and compared to untreated cells. Using information from public databases about molecular interactions, authors reconstructed a regulatory network linking the selected proteins and phosphoproteins, the investigated drugs targeting them and relevant cancer phenotypes. They translated the network into a computational model and trained it with the data produced and advanced algorithms for data inference. Using computer simulations of the computational model, they systematically predicted the response of the network to combinations of drugs and selected those combinations that in the simulations, at physiological doses, reverted the cancer phenotypes included in the model. For example, the simulations showed a strong G1-arrest when c-Myc inhibition was combined with inhibition of BRAF, MEK, and cyclin D1. This model prediction was experimentally validated when the RAFi-resistant melanoma cells where treated with combinations of JQ1, a c-Myc inhibitor, and a RAF inhibitor (PLX4032). Similar approaches have been used to look for drug combinations targeting the MAPK/AKT signaling network in colorectal cancer [77,78] and lung cancer [79].

Finally, computational models can be used to integrate information from different spatiotemporal levels. These models can integrate more detailed information about key molecular interactions between kinases, other signal mediators, and drugs into our understanding of the regulatory networks. In a study by Rukhlenko and co-workers, a computational model of the RAF/MEK/ERK signaling pathway in melanoma was constructed that integrated detailed molecular information about the thermodynamics and kinetics of the drug kinase interactions, quantitative information about protein expression levels, and data on relevant mutational and posttranslational modifications [67]. The computational model paid special attention to the dynamics of homo- and hetero-dimerization of RAF kinases. The computational model simulations predicted that two inhibitors with given, structurally different features targeting the same kinase but in different conformations could overcome a special type of RAF therapy resistance, which is mediated via paradoxical kinase activation by the drugs. This predictions were verified in in vitro experiments with A375 melanoma cells subjected to treatment with the two BRAF inhibitors sorafenib and B0R (2,6-difluoro-N-(3-methoxy-2H-pyrazolo[3,4-b]pyridin-5-yl)-3-[(propylsulfonyl) amino]benzamide) at different doses, alone or in combination.

Computational models can also be used to connect the (de)regulation of intracellular networks to the dynamics of tumor cell populations. In an own study, we established a multi-level computational model accounting for the resistance of melanoma cells to chemotherapy [70]. To this end, we reconstructed and modelled a melanoma-related regulatory network including growth factor receptors, TFs, miRNAs, and transcriptional targets. This network was connected to a computational model describing the dynamics of melanoma cell populations under control, single and combined genotoxic and cytostatic therapy. The computational model predicted that melanoma cells could become insensitive to both cytostatic and genotoxic drugs when displaying a signature of combined high expression for the E2F1 TF, low for miRNA-205 and high expression for the ERBB3 receptor, three of the molecular factors included in the intracellular regulatory network of the model. Interestingly, using transcriptomic profiling of tumor samples, Alla and co-workers showed that E2F1 overexpression is linked to melanoma aggressiveness, and this E2F1 over expression can induce oncogenic amplification via a positive feedback loop involving increased phosphorylation of ERK signaling [80]. Furthermore, in the above mentioned own study, computer simulations suggested, and in vitro experiments confirmed, that in genetically heterogeneous melanoma tumors, genotoxic drug treatment favors selection of chemoresistant clones by amplifying the deregulation of the selected gene circuits, such as the E2F1-p73/DNp73-miR-205 circuit [75]. These results suggest that tumor heterogeneity can promote resistance to anticancer therapy which has important consequences for the efficacy of therapies. Tumor heterogeneity is a feature that can be investigated using the recently developed single-cell sequencing techniques. In an own study, we employed single-cell RNA-seq to individually profile several hundreds of tumor cells taken from biopsies of three patients with different genetic backgrounds regarding *BRAF and NRAS*. The clustering of the cells in terms of their individual expression profiles was used to identify subpopulations of tumor cells with distinctive regulation for molecular circuits related to several melanoma-related phenotypes [81]. Single-cell analysis will in future be employed to analyze mechanism of treatment resistance present in individual cells or subclones.

## 7. Summary and Outlook

Targeted treatment of metastatic melanoma using small molecule inhibitors directed against mutated BRAF kinase or downstream kinases such as MEK1/2 is currently a mainstay of melanoma therapy. Although promising regarding initial treatment responses, secondary treatment resistance is currently a major challenge. The mechanisms of treatment response and resistance are investigated in more detail and have already provided deeper insights into the underlying molecular mechanisms. However, many unanswered questions have to be addressed in the near future, regarding the impact of clonal selection in tumors, the interaction between different signaling pathways, epigenetic mechanisms of treatment resistance, and the interaction between genome, transcriptome, and epigenome. These questions require a more holistic view of tumor growth and the role of regulatory pathways.
